# Wearable Battery‐Free Electrotherapy of Smartsensors for Wound Healthcare

**DOI:** 10.1002/advs.202519520

**Published:** 2026-03-25

**Authors:** Wenrui Zhang, Qian Lin, Jiancheng Zhang, Hao Mo, Wencheng Li, Yicheng Hu, Hongting Ma, Dan Zhao, Hongnv Yu, Nan Zhu

**Affiliations:** ^1^ Central Hospital of Dalian University of Technology School of Chemistry Dalian University of Technology Dalian Liaoning China; ^2^ Central Laboratory of Xinhua Hospital of Dalian University Affiliated Xinhua Hospital of Dalian University Dalian China; ^3^ Laboratory of Organic Electronics Department of Science and Technology Linköping University Norrköping Sweden

**Keywords:** wearable sensors, wound sensors, electrotherapy, supercapacitor‐powered

## Abstract

The design of a wearable bioelectronic device for electrotherapeutic wound healing and real‐time monitoring is critical for smart healthcare. However, developing multifunctional materials remains challenging due to energy supply or sensing interface issues. Herein, a simple strategy for integrating wound dressings of battery‐free electrotherapy and wound sensors via Dopamine (DA)‐modified MXene‐silver nanowire (Ag NWs)‐bacterial cellulose (BC) (PMAB) cross‐linked interpenetrating networks has been presented. Specifically, DA and BC significantly enhanced the antioxidant and mechanical properties of MXene, while Ag NWs improved the electrical and antimicrobial activities of PMAB. The solid‐state supercapacitor fabricated upon PMAB displayed excellent energy storage properties (2.5 F cm^−2^), replacing conventional power for delivering electrical stimulation (ES) to accelerate wound healing. NIH 3T3 fibrolast showed rapid migration and higher proliferation rate (over 70%) under ES (1 V). Meanwhile, wound dressing of cross‐linking interpenetrating structure of MXene and BC performs superior mechanosensing properties, with internal resistance change only 1.5 times of initial resistance over 60 days, which enables monitoring physical signal stabilization for wound assessment and management. This work would provide novel ideas of smartsensors for designing battery‐free wearable wound dressings.

## Graphical abstract

Herein, we propose a simple strategy for integrating battery‐free electrotherapy wound dressings with wound sensors. Solid‐state supercapacitors constructed by PMAB with cross‐linked interpenetrating network provide energy for electrotherapy. Meanwhile, excellent mechanical sensing performance of wound dressing was used for wound assessment. The study offers reference for design and application of wearable smart sensor wound dressings.

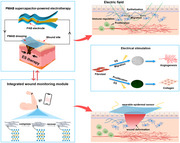

## 1 Introduction

As skin injuries increasing, wound management is critical for clinical care, particularly for chronic wounds, generally requiring extra interventions to promote healing [1, 2, 3]. Medical technology has presently delivered cutting‐edge therapeutic strategies for wound treatment, including hyperbaric oxygen therapy [4], electromagnetic therapy [5], ultrasound [6], photothermic therapy [7], negative pressure therapy [8] and ES [9, 10, 11]. ES with properties of non‐invasive, low side effects and simple operation, has emerged essential for wound care as it mimics nature healing mechanism of endogenous electric fields (EF). ES induces Na^+^/K^+^ transport between tissues, stimulates generation and reception of growth factors, guides neuronal growth, and further promotes cell migration and proliferation [12, 13]. Typically, modalities available for ES include high‐voltage pulsed current, low‐intensity direct current, and high‐frequency alternating current electric fields, requiring bulky equipment, specialized operators, hospitalization of patients [14], as well as external power sources. Owning to rapid charge‐discharge characteristics, supercapacitors have recently gained widespread use as portable energy storage modules in various power supply system's, especially as energy sources for ES [15]. Additionally, the assessment of wound healing in clinical practice relies predominantly on intensive observation and complex biochemical analyses, lacking approaches to monitor physiological level and progress of wound repair [16, 17]. Given above problems for ES, it is imperative to develop battery‐free wearable smart wound dressings for replacing traditional wound electrotherapy and management.

The majority of wound dressings (e.g., hydrogels) lack electroactivity, which are unable to respond of physiological electrical signals at wound site or external ES during wound healing process [18]. As a novel 2D nanomaterial composed of transition metal complexes, MXene have been extensively exploited in biomedical applications owing to metallic conductivity, energy storage, excellent hydrophilicity and abundant surface chemistry [19, 20, 21]. MXene nanosheets could accelerate wound healing by promoting wound angiogenesis. However, mechanical and electrical properties of scaffolds were not significantly enhanced, greatly limiting application in wound status monitoring, such as real‐time pressure sensing [22]. Additionally, poor environmental stability hinders long‐term application of MXene in biomedicine. BC is extensively employed as scaffold biomaterial for wound repair and regeneration in conventional medicine, owing to 3D fibrous porous microstructure, high water content (98%–99%), excellent hydrophilicity, good mechanical properties, and superior biocompatibility [23]. Recent years, modification of BC hydrogels with MXene has been investigated to manufacture BC‐based wound dressings for wound healing by ES, however, there exhibited poor antimicrobial properties at wound site [24]. Furthermore, pure MXene or bacterial cellulose, constrained by their inherent electrical and mechanical properties, is difficult to apply directly as wound healing dressings. Typically, there requires combination with conductive materials, bioactive substances, gels, and other medias to promote effective wound healing through synergistic effects [25, 26, 27].

Herein, wearable supercapacitor‐powered wound dressing, consisting of interpenetrating network of (polydopamine) pDA‐modified MXene and Ag NWs‐doped BC crosslinked by hydrogen bonding, has been reported. The tailored dressing exhibits superior antimicrobial, biocompatible, and mechanical properties. Specifically, modification of pDA surface with MXene enhances antioxidant properties without sacrificing capacitive performance. Additionally, addition of Ag NWs improves antimicrobial properties and conductivity. As‐fabricated supercapacitors provided external ES energy for generating effective EF to aid cell proliferation and differentiation, accelerating wound healing. Meanwhile, EF could induce directional cell movement and ordering, which facilitates wound healing. Moreover, healing process could be real‐time monitored by sensing properties of PMAB dressing via changes in resistivity. Prospectively, wearable battery‐free electrotherapy patch would provide promising strategy for wound healing and real‐time wound management.

## 2 Results and Discussion

### 2.1 Design of Battery‐Free Wearable Wound Patch for Electrotherapy

**FIGURE 1:**
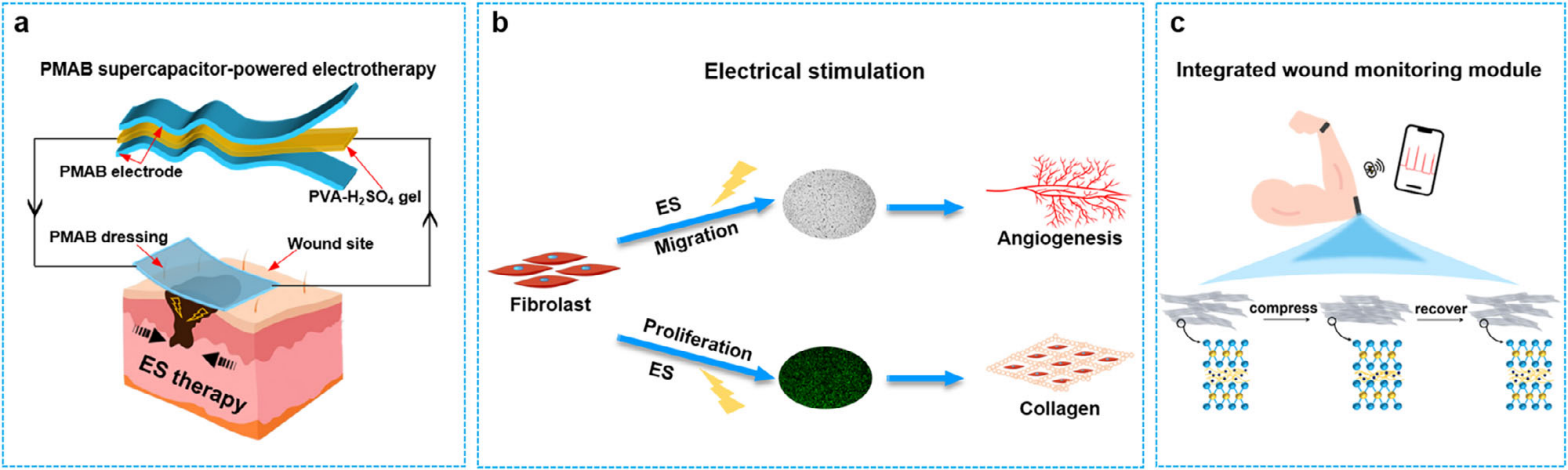
SCCs‐integrated wearable bioelectronic patch for electrotherapy. (a) Schematic diagram of the patch. (b) Effect of electrochemical stimulation on the proliferative phase of wound healing process. (c) Principles of integrating wearable epidermal sensors for effective wound monitoring.

MXene, 2D transition metal carbide and carbon‐nitride, has been extensively utilized as capacitive electrode for neural interfaces, owing to metallic conductivity and large specific surface area [28]. However, MXene‐based wound dressings have low environmental stability and mechanical strength, limiting effective electrotherapy and practical applications [29, 30, 31]. In order to improve stability and mechanical strength, oxidation‐resistant and mechanically stable MXene‐based wound dressing was designed. Initially, a protective layer of pDA was anchored on MXene through weak interactions between DA and polar groups of MXene surface, preventing restacking and oxidation of MXene nanosheets. Furthermore, the BC network was introduced to form extensive H bonds with MXene and constructed crosslinked network structure achieved better mechatronic properties for skin. Meanwhile, doped Ag NWs could compensate for insufficient electrical conductivity of BC. The resulting wound healing patch for battery‐free electrotherapy consists of PMAB‐based symmetric solid‐state supercapacitor (SCCs) and wound dressing. Apart from inherent energy storage properties of MXene, Ag NWs further enhances electrical storage capability for wide output voltage. When electrically active wound dressing was addressed over wound area, pre‐charged supercapacitor drives a current flowing through the dressing and generate exogenous EF (Figure 1a).

The wound dressing is expected to provide electrotherapy in three ways. First, EF promotes migration and proliferation of fibroblasts in the wound, accelerating tissue regeneration (e.g., formation of new epithelium, granulation tissue, and blood vessels) (Figure 1b). Second, dressing electrodes could magnify the stimulation effect by injecting charge efficiently into the skin through bioelectronic interface [32]. Third, Ag NWs in dressing offers favorable antimicrobial properties, preventing further infection of wound and promoting effective healing. Moreover, benefited from cross‐linking and interpenetrating network structure of MXene with BC, impedance variation caused by MXene layer spacing enables real‐time monitoring of wound deformation, and thereby realizes wound healing management (Figure 1c).

**FIGURE 2:**
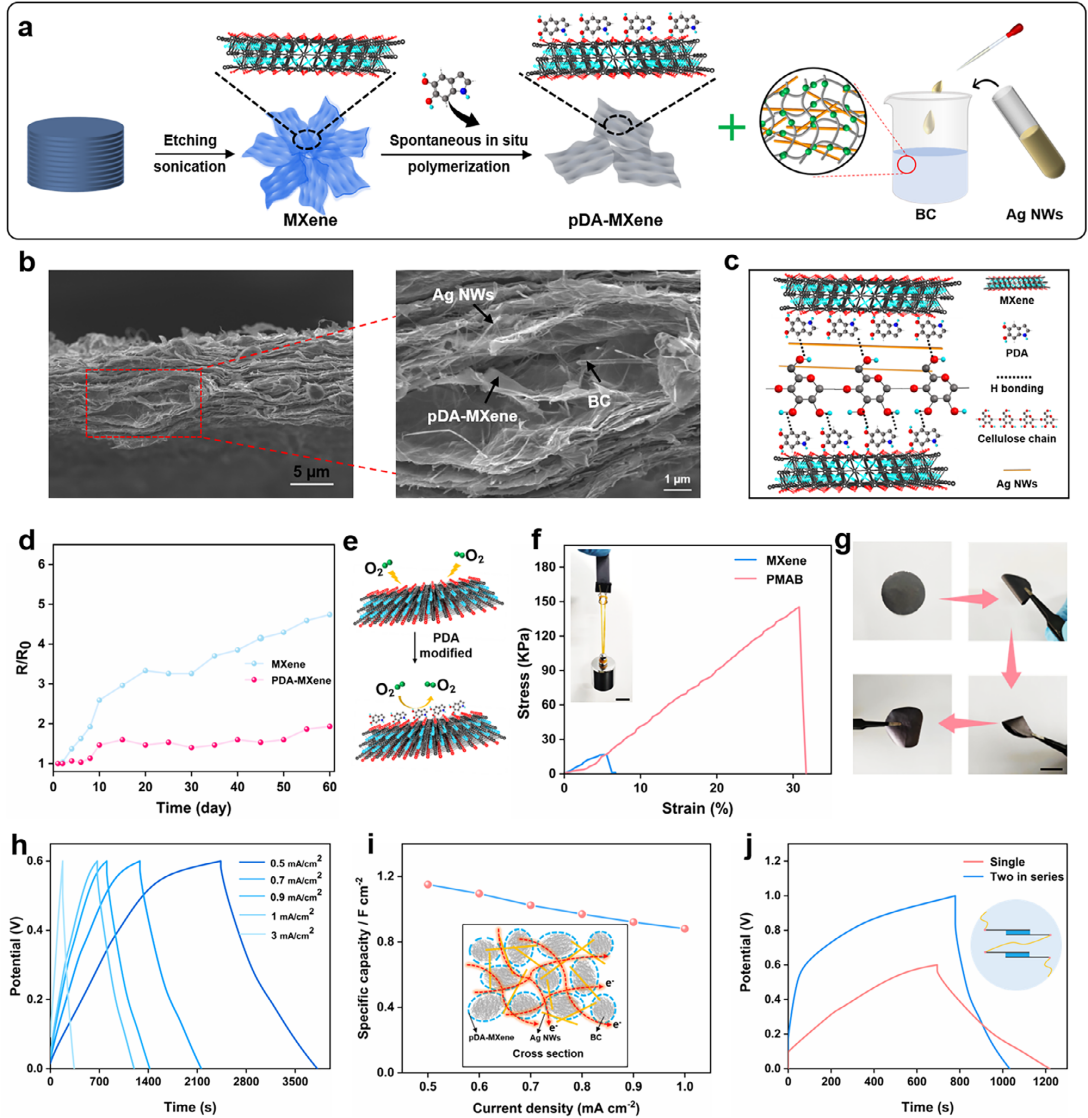
Characterization of PMAB film. (a) Schematic diagram of PMAB preparation process. (b) Cross‐sectional SEM image of PMAB film. (c) Structure of PMAB cross‐linked interpenetrating network. (d) The change of the electrical resistance of MXene and PMAB films over time. (e) Proposed binding mechanism of pDA on MXene. (f) Tensile stress–strain curves of MXene sheets and PMAB film. Scale bar: 1 cm. (g) Bending performance of PMAB. Scale bar: 2 cm. (h) GCD profiles of SCCs with different charging‐discharging currents. (i) Specific capacitances at different current densities of SCC a scan rate between 0.5–1.0 mA cm^−2^. Inset: Schematic illustration of electron migration in PMAB. (j) GCD curves of single SCCs and two SCCs in series.

The performance of the supercapacitor as wearable energy supply was evaluated. Initially, successful incorporation of dopamine into MXene was confirmed through scanning electron microscopy (SEM), atomic force microscopy (AFM), X‐ray diffraction (XRD), and Fourier transform infrared spectroscopy (FT‐IR). The interlayer spacing of MXene was effectively expanded, thereby improving the ion transport pathways and subsequently enhancing electrical performance (Figures S1 and S2). Subsequently, composites containing pDA‐MXene and BC‐Ag nanowires (PMAB) with a thickness of 27 ± 2 µm were prepared using a one‐step physical self‐assembly technique (Figure 2a; Figure S3). Cross sectional SEM image of PMAB film illustrated cross‐linked interpenetrating structures of PMXene and Ag NWs‐BC, resulting in durable layered network morphology (Figure 2,c; Figures S4 and S5), while pure MXene exhibited only a layer structure without a network (Figure S6). Moreover, surface pores of PMAB are densely and uniformly distributed, with a statistically determined pore size of approximately 0.6 µm (Figure S7). The XRD patterns further confirmed the PMAB structure. Selective etching of Ti_3_AlC_2_ MAX phase appeared shift from 9.5° to 6.3° of (002) peak, validating successful fabrication of MXene flakes [33]. Apart from MXene characteristic peaks, Ag (111) peak of 38.02° was also distinctly observed in PMAB, demonstrating that Ag NWs interpenetrated well across MXene and BC network structures [34]. Moreover, cellulose (002) of 22.66° decreases gradually, suggesting the encapsulating effect of MXene and Ag NWs on the cellulose filaments (Figures S8 and S9) [35]. The Raman spectra of PMAB and MXene exhibited similar peaks centered at 201, 282, 389, 519, and 720 cm^−1^ (Figure S10). The first three peaks were primarily attributed to A_1g_ and E_g_ symmetry vibrations of Ti and O, respectively, and the last two peaks were assigned to E_g_ and A_1g_ symmetry vibrations of C atoms [36]. Subsequently, the mechanism of oxidative self‐polymerization of DA in preparation of PMAB was evaluated by X‐ray photoelectron spectroscopy (XPS). The catechol moiety in DA is generally recognized strong binding affinity with multivalent metal ions, and combination with titanium oxide via ligand bonding was intensively explored [37]. XPS spectra verified presence of C, N, O, Ti, F and Ag elements in PMAB (Figure S11). The deconvolution C 1s spectrum of PMAB revealed that C─Ti peak shifted to lower binding energy side, suggesting that electrons were possibly diverted from pDA to MXene. XPS O 1s spectra indicated presence of quinone state (C─O), which overlapping with C─O─Ti bonding peaks. Additionally, the binding energies of Ti─O and C─Ti─Mx (M for O and OH) were slightly weakened, attributed to interfacial electron transfer arising from ligand dehydration of ─OH groups on the surface of catechol and MXene (Figure S12). The Ti 2p spectra displayed minor decrease in the binding energy, demonstrating an increase in the electron cloud density at the MXene surface in PMAB. Furthermore, the peaks at 399.5 eV (R─NH─R), 397. 7 eV (R = N─R), and 394.6 eV (N─Ti bond) in XPS N 1s spectrum of PMAB showed oxidative polymerization of DA, and then binding to the MXene surface (Figure S13). The high‐resolution Ag 3d spectra of PMAB revealed distinct fitted peaks, suggesting that Ag NWs were embedded in the MXene and BC interpenetrating structures (Figure S14) [38].

Motivated by the above results, DA oxidative polymerization scheme for functionalized MXene was proposed (Figure S15). DA was oxidized to form dopamine quinone by dissolved oxygen and formed thin pDA monolayer on the surface of MXene after successive cyclisation, oxidation, isomerization, auto‐polymerization and coordination polymerization. The resistance of MXene and pDA‐MXene films for 60 days was illustrated (Figure 2d). After 60 days, the resistance of pDA‐MXene films remained stable (only 1.5 times), while pure MXene films increased by nearly 5 times, resulting from polydopamine layer strongly bonding to the surface of MXene sheet and blocking the penetration path of oxygen permeation (Figure 2e). The maximum tensile strength of PMAB is 145 KPa, approximately 9 times than pure MXene, which could be employed to raise up 1000 times of own weight (Figure 2f). Thus, there demonstrates reliable mechanical properties of as‐fabricated PMAB (Figure 2g).

Wearable systems with miniaturized energy harvesting and storage devices are in high demand to facilitate battery‐free operation [39, 40, 41]. PMAB‐based SCCs were developed to power ES upon the favorable electrochemical properties of PMAB. Cyclic voltammetry (CV) and galvanostatic charging‐discharging (GCD) curves of PMAB showed double layer capacitance and high‐rate behavior, achieving high capacitance of 2500 mF cm^−2^ at 5 mA cm^−2^ (Figure S16). Capacitive and diffusion capacitance contributions were evaluated as the following equation [42]:

(1) $$i\;=k_{1}\;v+k_{2}v^{1/2}\;$$

where k_1_ and k_2_ are constants, i is the current at a given potential V, v is the scan rate (V s^−1^). k_1_v and k_2_v^1/2^ are the fractions of capacitive contribution and diffusion contribution, respectively. The contribution ratios of diffusion and capacitive control are quantitatively determined (Figure S17).

The fraction of surface‐control capacitance increases from 40% to 80% when scanning rate increases from 10 to 70 mV s^−1^, resulting in the predominance of surface electrochemical activity by PMAB. Moreover, there displayed cycling stability with capacitance retention of 84% after 5000 cycles (Figures S18 and S19). Additionally, electrochemical storage behavior of MXene, pDA‐MXene, MXene‐BC, and pDA‐MXene‐BC was investigated (Figures S20–S23). Apparently, PMAB achieves longer symmetric triangular discharge time and negligible voltage drop (Figure S24), implying superior dynamic reversibility and high capacitance, which maintained steady resistance even after 5000 cycles (Figure S25). It is notable that the capacitive performance of MXene was unaffected by the functionalization of pDA, further confirming that pDA only forms protective layer on MXene surface for prevent oxidation. To further explored energy module for accommodating ES wound healing, symmetric all‐solid‐state SCCs based on PMAB was constructed (Figure S26a). Excellent cycling reversibility and cycling stability of PMAB‐based SCCs was demonstrated by CV (Figure S26b), GCD (Figure 2h) and cycling test profiles (Figures S27 and S28). Additionally, robust encapsulation ensures that components within the device are difficult to leak into the environment, demonstrating excellent safety performance. (Figure S29). The interpenetrating networks of MXene and BC conductive drastically decreased charge/ion transport paths and accelerated charge/ion transfer rate (inset of Figure 2i), with specific capacitance up to 1200 mF cm^−2^ (Figure 2i). By connecting two SCCs in series, the voltage window was simply scaled up to 1 V (Figure 2j), allowing for effective external EF stimulation for wound healing.

### 2.2 Electrical Stimulation Facilitates Cell Behavior and Biocompatibility Assessment

**FIGURE 3:**
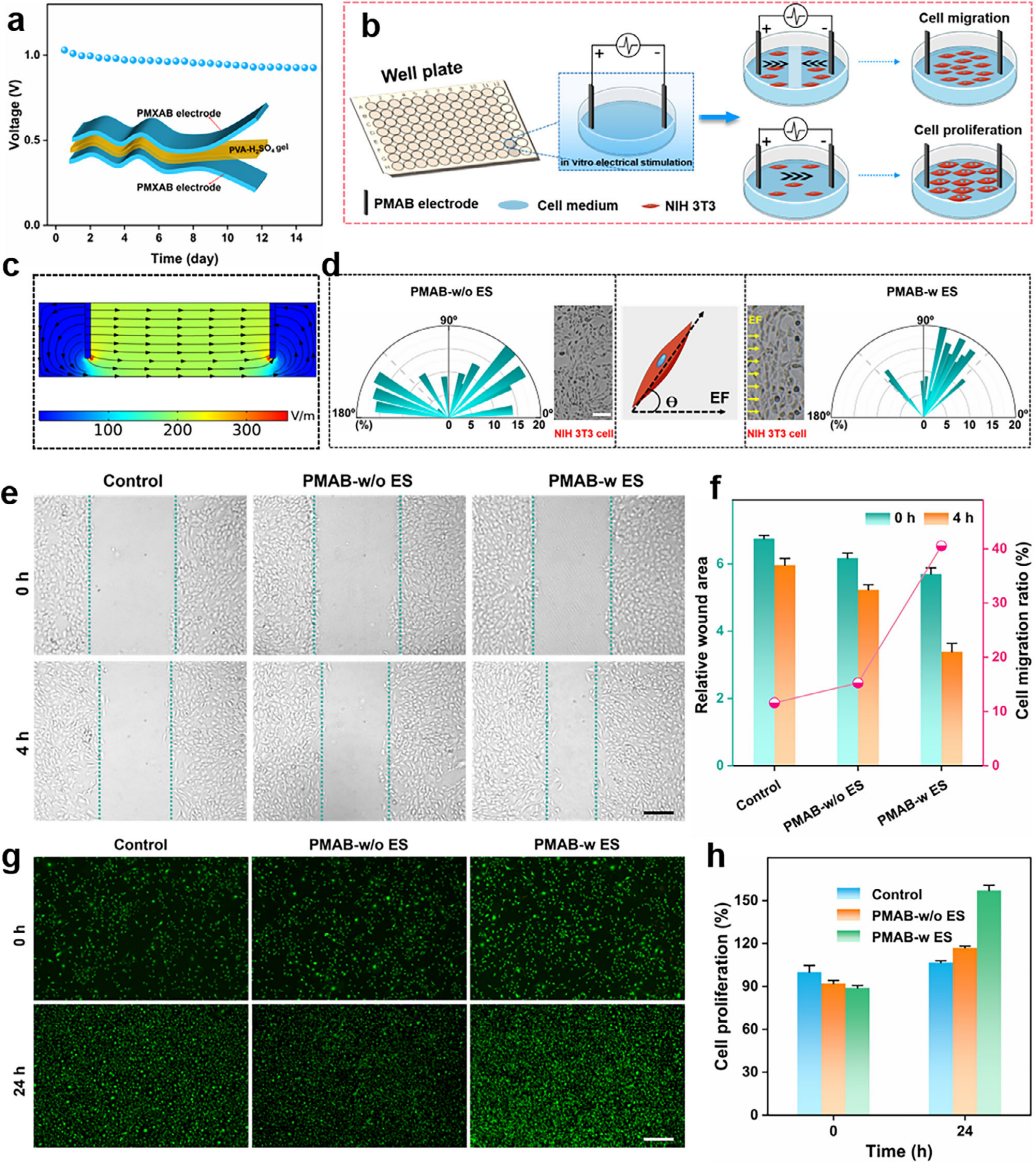
In vitro biocompatibility with ES evaluation of PMAB. (a) Voltage stability curve of SCC over 14 days. (b) Effect of ES on cell proliferation and migration. (c) Electric field distribution simulated by directional coupling. (d) NIH 3T3 cell images and quantification of cell angles in the PMAB‐w/o ES group and PMAB‐w/ ES group after 24 h. Results are plotted as means ± SD (*n* = 3). Scale bar: 50 µm. (e) Cell migration images of fibroblasts after applying ES of 1v for 4 h. Scale bar: 100 µm. (f) Corresponding cell migration ratio of fibroblasts. (g) Fluorescence images of fibroblasts after ES recorded on days 0 and 1. Scale bar: 100 µm. (h) Cell proliferation ratio of fibroblasts.

Cell proliferation and migration are key processes in wound healing. Fibroblasts engage in migration, proliferation and degradation of fibrin clots in wound healing and produce new extracellular matrix (ECM) components and assorted cytokines [43]. The proliferative and migratory impact of ES from SCCs on fibroblasts was investigated in vitro. Firstly, long‐term stability of voltage generated by supercapacitor was confirmed by maintaining 89% over 14 days, which was capable of delivering a stable external supply for ES (Figure 3a). In typical experimental setup, two PMABs were coupled to the positive and negative poles of SCCs power supply for providing stable voltage (Figure 3b). The self‐discharge curve shows that PMAB‐based SCCs can maintain a voltage exceeding 0.74 V for over 0.5 h (Figure S30). Thus, PMAB‐based SCCs with excellent self‐charging performance are suitable for driving electrical stimulation therapy. Electrochemical communication between living cell and cell involves Faraday processes, ion fluxes, potential gradients, and chemical releases [44]. EF distribution in cell culture medium was first simulated by electrochemical modeling to assess the effects of ES on cell function (Figure 3c). Coupled SCCs generated EF intensities of 200 mV mm^−1^ in the center of culture plate, and the resulting directional EF plays a crucial role in the modulation of cellular behaviors, including cell‐cell junctions, orientation of cell division, and cell migration trajectories (electro‐axial or electro‐axial) [45].

In the PMAB‐w/o ES group, cells were randomly distributed throughout the growth phase, with an orientation angle of ‐0.02 ± 0.04. In contrast, cells in the PMAB‐w/ ES treated group displayed ordered growth pattern, with angle of ‐0.62 ± 0.05, suggesting that cells were predominantly vertically aligned (Figure 3d). The EF‐induced alignment could be attributed to the reorientation of the cytoskeleton through Wnt/β‐catenin and PI3K/Akt signaling pathways during mitosis [46]. Subsequently, cell migration experiments were performed to investigate the influence of PMAB wound dressing on cell migration, with voltage of 0.5 V and 1 V, respectively (Figure 3e; Figure S31a). It is similar to the voltage range (0.5–1.5 V) of employing percutaneous or direct tissue electrical stimulation to promote wound healing in previously reported research [9, 10], indicating that the voltage range is both effective and widely applied. The results revealed that cell migration rates of PMAB‐w ES‐1 V were twice compared to those of control and PMAB‐w/o ES groups (Figure 3f;Figure S31b,c), owning to directional migration enabling cellular contractility toward the scratch area, thereby accelerating wound closure [47].

Moreover, PMAB‐w/ ES (1 V) group demonstrated faster closure than PMAB‐w/ ES (0.5 V) group. Under optimal conditions (1 V, 0.5 h), live‐dead cell staining was performed for fibroblast proliferation assay to assess biocompatibility of PMAB. After co‐culture for 24 h, all three groups of fluorescent staining images demonstrated promoted properties on cell proliferation (Figure 3g). Notably, cell proliferation rate in PMAB‐w/ ES group was significantly superior to control and PMAB‐w/o ES groups by exceeding 70% (Figure 3h). Furthermore, it is of great significance for human health monitoring with antibacterial properties. The antibacterial performance of PMAB against Gram‐positive (*S. aureus*) (Figure S32a) and Gram‐negative (*E. coli*) (Figure S32b) bacteria was investigated. After incubating PMAB with bacteria for 24 h, the test group exhibited inhibition of *E. coli* and *S. aureus* over 80% and 75%, respectively, while there was no obvious inhibitory result in control group. It is attributed to the ability of Ag NWs possessing broad‐spectrum and long‐lasting antimicrobial properties, enhancing antimicrobial performance of PMAB dressings [48]. In vitro hemolysis test demonstrated that the hemolysis rate of erythrocyte suspensions incubated with target materials was less than 5 (Figure S33). Based on above good biocompatibility, PMAB‐based electronic components were expected as sensing systems for human health monitoring.

In tissue reconstruction process, over‐deformation, tearing or stretching at the wound site could make neoplastic tissue damage, subcutaneous capillary rupture or edema [49]. Thus, there is a necessity for real‐time monitoring of wound strain. Considering superior combination of resilience, antimicrobial properties and mechanical strength of PMAB, it was able to detect subtle variation in wound tissue deformation or complex body movements (Figure S34). The sensing mechanism of PMAB has been demonstrated (Figure S35a). Specifically, MXene and BC form a network cross‐linking structure with H bond, and the distance between neighboring interlayers would decrease under external pressure, causing resistance to decrease [50].

When repeated pressing and releasing of finger was conducted on the wearable PMAB epidermal pressure sensor, there received stable response (Figure S35b). Attaching to wrist (Figure S35c) and elbow (Figure S35d), PMAB‐based wearable epidermal sensors could monitor signals generated by joint movements. Moreover, characteristic signals of swallowing revealed great potential of PMAB wearable sensors for physiological monitoring (Figure S35e). Therefore, wearable PMAB‐based epidermal pressure sensors would be ideal for the exploitation of health monitoring systems.

### 2.3 Battery‐Free Electrotherapy of Wearable Smartsensors for Wound Healthcare

**FIGURE 4:**
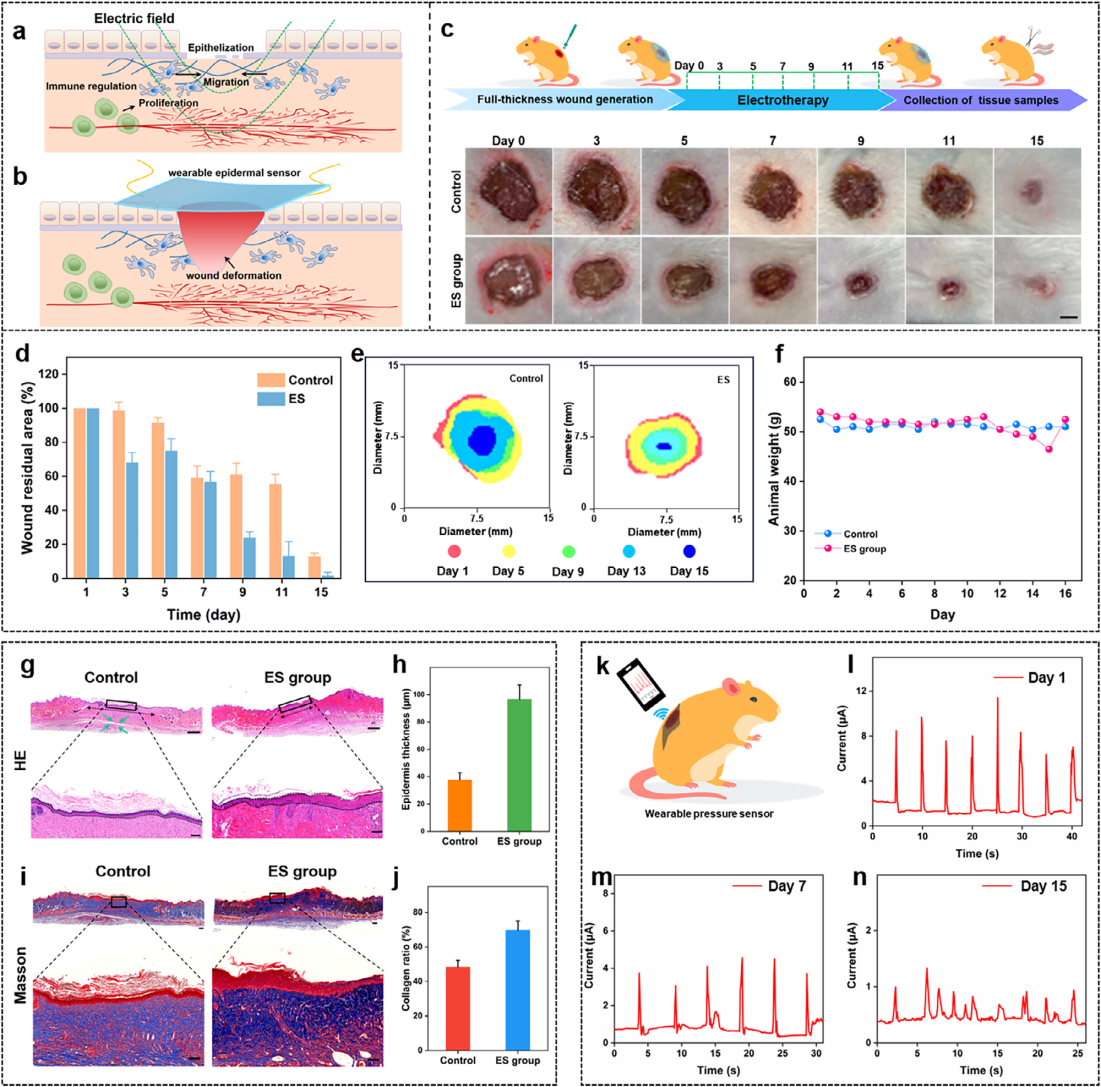
In vivo evaluation of wound healing efficacy and epidermal pressure monitoring. (a) Integrated application schematic diagram. (b) The effect of EF on tissue regeneration. (c) Wound healing in different treatment groups throughout a 15‐day period. Scale bar: 2 mm. Statistical analysis of wound area (d) and schematic diagrams (e) on days 1, 3, 5, 7, 9, 11, and 15 in different treatment groups. (f) Changes in weights of rats in different groups. Histological evaluation of wound tissues. H&E staining (g) and quantitative results (h) of wound epidermal thickness on day 15. Masson's staining (i) and dermal collagen density (j) of wounds on day 15. Scale bar: 100 µm. (k) The rat model for wireless wound monitoring. Wearable epidermal sensors based on PMAB for monitoring wound deformation at day 1 (l), day 7 (m) and day 15 (n).

For practical application of battery‐free electrotherapy, wearable dressings were employed directly for in vivo wound healing. The applied electric field could accelerate cell differentiation and migration, promoting wound epidermal regeneration (Figure 4a). Meanwhile, integrated wearable epidermal sensors monitors wound deformation in real‐time for wound management (Figure 4b). The dressing was placed on full‐thickness circular wound (8 mm diameter) on the back of rat. ES test (1 V, 30 min) was performed by SCCs power supply by ends of dressing. Wound area of supercapacitor‐powered electrotherapy was in‐site recorded over period of 15 days during healing process (Figure 4c). Compared with control group without electrotherapy of remaining would area 61% after 9 days, ES‐treated wounds gradually reduced into 24% along EF direction. After healing treatment of 15 days, electrotherapy wound was fully recovered (≤ 2%), while control group was still 15% (Figure 4d). Meanwhile, the wound simulation graphs visualize changes in wound size during 1–15 days for two treatment groups (Figure 4e), indicating faster recovery rate with electrotherapy. Moreover, the weight of rat fluctuated within normal range during healing process (Figure 4f). Furthermore, negligible swelling properties confirm that PMAB maintains excellent electrical and mechanical performance in wound exudate environments (Figure S36).

The results revealed that wound healing under supercapacitor‐powered dressing conditions was significantly superior over 15‐day period. Moreover, ES voltage could be easily adjusted by battery‐free electrotherapy upon practical size or acute wound. To assess histological changes during healing process, wound tissues were harvested and stained with hematoxylin/eosin (H&E) or Masson on wound site of day 15. The H&E staining images revealed the existence of scab (green arrow) on wound area for the control, whereas scab formulation was not obvious for ES group (Figure 4g). Additionally, ES group displayed epidermal integrity (black mark) around regenerated tissues, with 2.6 times thicker than control group (Figure 4h).

Meanwhile, the deposition of neoplastic collagen in regenerated skin tissues was performed by Masson staining, which is an important indicator of tissue remodeling [51]. The density of collagen fibers increased in wounds area of ES group (black mark) (Figure 4i), while limited deposition in control group. Moreover, collagen fibers in ES group were more delicately arranged, effectively inhibiting the formation of loose collagen fiber structures [52]. Quantitative analysis indicated that PMAB‐based ES increased collagen deposition by approximately 1.4 times compared with control group (Figure 4j). The synergistic effect of PMAB‐based wound dressing and battery‐free ES could effectively promote wound re‐epithelialization, granulation tissue formation and collagen deposition, thereby promoting skin tissue regeneration.

Additionally, wound bed strain is an important parameter in wound assessment with high sensitivity to swelling and dehiscence [53]. Wound strain could cause secondary tears or even delay the healing process by preventing the growth of wound tissue and cells. Thus, continuous on‐site monitoring of the wound site is essential for skin restoration. Wound strain monitoring in rat was further investigated by PMAB‐based wearable epidermal smartsensors (Figure 4k). The wound strain response was recorded for various days (day 1, day 7 and day 15) (Figure 4l,n). As the wound gradually healed, the signal gradually decreased. The state of wound healing can be monitored in real‐time by strain signal variation through a smartphone.

## 3 Conclusion

In summary, battery‐free wearable smart dressing based on PMAB films was demonstrated for ES wound healing and epidermal bioelectronics monitoring. Modified MXene displayed promising antioxidant and mechanical properties, while doped Ag NWs significantly strengthened electroactivity and antimicrobial properties (*E. coli*: 80%; *S. aureus*: 75%) of wound dressing. Benefiting from cross‐linking and interpenetrating network structure of PMAB, supercapacitor module displayed superior electrical output characteristics (1 V) during healing period. Therapeutic experiments (in vivo and in vitro) demonstrated that ES generated by supercapacitor significantly stimulated cell proliferation (over 70%), migration, and angiogenesis of wound, resulting in promotion of collagen deposition and wound healing. Meanwhile, PMAB was sensitive to subtle movements for employing as epidermal pressure sensor, accurately acquiring real‐time signals of wound deformation by smartphones. Prospectively, as‐designed battery‐free wound dressing is facile for electrotherapy, providing ideas for wound repair and smartsensing healthcare.

## Conflicts of Interest

The authors declare no conflicts of interest.

## Supporting information




**Supporting File**: advs75016‐sup‐0001‐SuppMat.pdf.

## Data Availability

The data that support the findings of this study are available from the corresponding author upon reasonable request.
