# Supplementary material for: Wearable Battery‐Free Electrotherapy of Smartsensors for Wound Healthcare
Source: Adv Sci (Weinh). 2026 Mar 25;13(32):e19520. doi: 10.1002/advs.202519520 (PMC13252601; doi:10.1002/advs.202519520)
Supplement: Supplementary file 1 — Supporting File: advs75016‐sup‐0001‐SuppMat.pdf. [file ADVS-13-e19520-s001.pdf]

Supporting Information for

**Wearable Battery-free Electrotherapy of Smartsensors for Wound Healthcare**

*Wenrui Zhang[1,§], Qian Lin[2,§], Jiancheng Zhang[1], Hao Mo[1], Wencheng Li[1], Yicheng Hu[1], Hongting Ma[1], Dan Zhao[3,*], Hongnv Yu[2,*], and Nan Zhu[1,*]*

[1]Central Hospital of Dalian University of Technology, School of Chemistry, Dalian University of Technology, Dalian, Liaoning, 116024, China
[2]Central Laboratory of Xinhua Hospital of Dalian University, Affiliated Xinhua Hospital of Dalian University, Dalian, 116021, China
[3]Laboratory of Organic Electronics, Department of Science and Technology, Linköping University, 60174 Norrköping, Sweden

W.R. Zhang, J.C. Zhang, H. Mo, W.C. Li, Y.C. Hu, H.T. Ma, Prof. N. Zhu.

Central Hospital of Dalian University of Technology, School of Chemistry, Dalian University of Technology, Dalian, Liaoning, 116024, China

E-mail: nanzhu@dlut.edu.cn (N. Zhu)

Q. Lin, Prof. H.N. Yu

Central Laboratory of Xinhua Hospital of Dalian University, Affiliated Xinhua Hospital of Dalian University, Dalian, 116021, China

E-mail: hongnvyu2312@sina.com (H.N. Yu)

Prof. D. Zhao

Laboratory of Organic Electronics, Department of Science and Technology, Linköping University, 60174 Norrköping, Sweden

E-mail: dan.zhao@liu.se (D. Zhao)

[§]W.Z. and Q.L. contributed equally.

**This PDF file includes:**

Supplementary Text

Figure S1 to S28

## Experimental Section

### Materials and Reagents

Dopamine hydrochloride (DA), concentrated sulfuric acid ($H_2SO_4$), hydrogen peroxide ($H_2O_2$, 30%), and concentrated hydrochloric acid (HCl) were obtained from Bonuo Biochemical Reagent Co., Ltd. (Dalian, China). Poly (vinyl alcohol) (PVA) was obtained from Aladdin (Shanghai, China). $Ti_3AlC_2$ MAX was purchased from Forsman Scientific (Beijing) Co., Ltd. and LiF and ethanol. Bacterial nanocellulose (BC, 0.75 wt %) was supplied by Guilin Qihong Technology Co., Ltd. (Guilin, China). Silver nanowires (Ag NWs) aqueous suspension (15 mg $ml^{-1}$, in DI water) was purchased from Shanghai Ouyi Organic Optoelectronic Material Technology Co., Ltd. (Shanghai, China). Deionized water with a resistivity of 18.2 MΩ·cm was utilized in all experiments. All chemical reagents were of the available analytical grade and used as received.

### Synthesis of $Ti_3C_2T_X$ MXene and polydopamine-modified MXene

$Ti_3C_2T_x$ MXene was fabricated as described in a previous report (29). Initially, 1 g of LiF was dissolved in 20 ml of 9 M HCl. Subsequently, 1 g of $Ti_3AlC_2$ powder was incorporated into the mixed system under stirring. The mixture was maintained in an oil bath at 35 ℃ for a total of 24 hours. Then, the resulting suspension was washed to a pH close to 6 and centrifuged once more. Finally, the precipitate was sonicated for 1 hour and centrifuged at 3500 rpm for 1 h to yield a $Ti_3C_2Tx$ MXene nanosheet suspension. The MXene suspension was then freeze-dried for further use.

For polydopamine (PDA)-modified MXene, DA solution (2 mg $ml^{-1}$) was dropped into 10 ml of $Ti_3C_2Tx$ aqueous dispersion (2 mg $ml^{-1}$) at the appropriate mass ratio. The dispersion was then further stirred (200 rpm, 30 ℃) for 1 h and centrifuged (3500 rpm, 5 min) to remove unreacted monomers from the solution. The precipitate was

redispersed in deionized water using ultrasound to obtain a pMX dispersion.

**Preparation of the BC/Ag NWs dispersion**

The BC solution was obtained via dilution of the highly concentrated colloid. 20 g of BC colloid was dispersed in a beaker with DI to a concentration. Then, the solution was stirred for 30 minutes at room temperature to produce a homogeneous dispersion. Subsequently, certain amount of Ag NWs suspension (1 mg $mL^{-1}$) was added to the BC dispersion and stirred continuously for 30 minutes to obtain a uniform suspension.

**Preparation of pristine pMX-Ag NWs-BC film (PMAB)**

The PMAB film was prepared by vacuum-assisted filtration of the pMX and BC/Ag NWs dispersion through a cellulose filter film (0.45 μm in pore size), and naturally dried in air at 60 ℃. The MXene film, pMX film, BC film, MXene/BC film and pMX/BC film were prepared through a similar procedure. Furthermore, the prepared PMAB (1×2 cm) was integrated by polyimide tape and copper wires for electroactive dressing and pressure sensor fabrication.

**Fabrication of all-solid state symmetric supercapacitors**

A flexible PMAB paper was directly employed as an electrode. All-solid-state symmetric supercapacitors were manufactured utilizing PVA/$H_2SO_4$ as a gel electrolyte. Briefly, 6 g of PVA were added to 60 ml 1 M $H_2SO_4$ at 85 ℃ under continuous stirring for 3 h to completely dissolve the PVA, resulting in a transparent PVA/$H_2SO_4$ gel electrolyte. Copper tapes were attached to the ends of each electrode to provide an electrical connection. Two pieces of prepared composite paper were placed on either side of the electrolyte and encapsulated to assemble a flexible supercapacitor.

**Electrochemical measurements and sensor data collection**

Electrochemical characterization was carried out at room temperature using a PGSTAT302N potentiostat/galvanostat (Metrohm, Autolab B.V., Utrecht, The Netherlands; serial no.: AUT88992, manufactured in January 2016). The instrument is capable of ±10 V voltage range, current measurement accuracy of ±0.1%, and a maximum sampling frequency of 1 MHz. Cyclic voltammetry (CV) and electrochemical impedance spectroscopy (EIS) were conducted using the integrated Nova/GPES software for data acquisition and analysis. The prepared sample films were

employed as working electrodes (WE), while platinum plates and Ag/AgCl electrodes were used as counter electrodes (CE) and reference electrodes (RE), respectively. Meanwhile, 1 M $H_2SO_4$ was utilized as the electrolyte for electrochemical measurements. For sensor performance evaluation, signal feedback was measured by i-t curves at a voltage of 0.05 V. Additionally, an electrochemical analyzer was used to collect the sensing data. These volunteers have consented to demonstrate the self-powered monitoring system and to release the results to the public.

**Characterization**

The surface morphologies and microstructures were measured by Scanning electron microscopy (SEM), Transmission electron microscope (TEM) on JEM-F200 with an accelerating voltage of 200 kV. The X-ray diffractometer (XRD, Rigaku SmartLab 9KW, Bruker, Germany) with Cu Kα radiation (λ = 1.5444 Å, 40 kV and 40 mA) was used to measure the crystallization of the samples, and the range of diffraction angles (2θ) was from 5° to 70°. The elemental constituents and elemental states were analyzed by X-ray photoelectron spectroscopy (XPS, Thermo Fisher Scientific, USA) with a Thermo ESCALAB 250XI multifunctional imaging electron spectrometer using 150 W Al Kα radiation. Raman spectra were carried out using in Via Qontor with an excitation wavelength of 532 nm. The sample were cut into a rectangle (1 ×2 cm) and the tensile properties were measured in a universal testing machine (CMT-4503, Jinan Metz Test Technology Co., LTD) at a constant tensile speed of 5 mm $min^{-1}$. Film thickness characterization was performed using Electronic Digital Micrometer (AGL, 0-25 mm).

**Finite Element Analysis Simulation**

The distribution of EF and mean flux in cell culture plates was modeled using COMSOL Multiphysics software. Modeling was done in an electrolytic cell containing PMAB dual electrodes (0.2 mm thickness). The dimensions of the 2D model were set to 4 mm (electrode height) ×10 mm (two electrodes distance). The electric field is described by

$$\nabla \cdot D = \rho$$
$$E = -\nabla V$$

where D, ρ, E, and V denote the electric displacement field, charge density, electric

field, and electric potential.

The boundary condition for the computational domain was set by

$$n \cdot D = 0$$

where n indicates the normal to surface of the boundary. The potential of the anode was set to be 1 V, and the potential of the ground electrode was set to be 0 V.

**In vitro cell culture**

NIH-3T3 fibroblasts were purchased from the Cell Resource Centre of the Institute of Basic Medical Sciences, Chinese Academy of Medical Sciences, and cultured in l L glutamine-hyperglycemic medium (DMEM, gibco) with 10% calf serum (CCF) and 1% penicillin-streptomycin solution, and placed in a 37 ℃, 5% $CO_2$ incubator. Logarithmic growth phase cells in good condition were used for each experiment.

**Cell proliferation assay**

Cells were inoculated in 96-well plates at a density of $2 \times 10^3$ per well and cultured for 12 hours. The well plates were placed with two vertically aligned electrode materials to produce an ES effect. The electrodes were connected to the two terminals with a stimulation time of 30 min (1 V). The control group only placed the electrodes in the well plates without stimulation. The blank group was not treated in any way. Cell viability was detected by CCK-8 (Cell Counting Kit-8) assay at 0, 24, 48 and 72 h after stimulation, respectively. 10 μL of CCK-8 solution was added to each well, and the optical density (OD) of each well was measured at 450 nm wavelength after 1~2 h incubation using an enzyme marker to calculate the relative cell proliferation rate. For fluorescence imaging, cells co-cultured with control, PMAB-w/o ES, and PMAB-w/ ES were stained with 2 μM Calcein AM dye and directly observed under an inverted fluorescence microscope (DMIL, Leica).

**Cell migration assay**

Cells were inoculated in 6-well plates at a density of $1 \times 10^6$ per well and cultured for 12 h until 80% to 90% fusion. A 200 μL sterile pipette tip was used to create a gap in the middle of the plate and washed twice with PBS to remove cellular debris. Two vertically aligned electrode materials were placed in each well so that the direction of EF was parallel to the direction of cell migration. The electrodes were connected to the two

terminals controlled by a linear motor, and the output voltages were set to 0.5 V and 1 V. The control group only placed the electrodes in the cell wells without ES. The blank group was not treated in any way. The migration area was measured from the images using Image J software. The rate of migration was expressed as cell migration ratio, which is the ratio of the change in scratch area over different time intervals to the initial scratch area.

$$\text{cell migration ratio (\%)} = \frac{A_0 - A_t}{A_0} \times 100\%$$

where $A_0$ and $A_t$ are the areas of the initial and healed scratch areas at different time intervals, respectively.

Compared to the control group, the ES group should demonstrate a ≥30% increase in scratch healing rate within 24 hours and a ≥20% increase in proliferation rate.

**Strain and medium**

The experimental strains were obtained from clinical isolates of Escherichia coli and Staphylococcus aureus from the Department of Laboratory Medicine of Xinhua Hospital affiliated to Dalian University, which were identified by Micro Scan Walk Away40 Plus (Siemens, Germany), and then stored at -80 ℃ for spare use. Nutrient broth and MH broth medium (Hangzhou Tianhe Microbiological Reagent Co., Ltd.), nutrient agar medium (Zhengzhou Beret Biological Reagent Co., Ltd.).

**Hemolysis assay**

Nude mouse blood was diluted 10-fold with PBS to obtain red blood cells (RBCs), which were then centrifuged at 4 ℃ for 10 minutes (1500 rpm) to remove the supernatant and collect the erythrocytes. 200 μL of erythrocyte solution was added to 800 μL of PBS and then incubated with 200 mg of $H_2O$ (positive group), PMAB, and PBS (negative group) for 2 hours at room temperature. The sample solution was then centrifuged for 10 minutes (1500 rpm), and the absorptivity of the supernatant was measured using UV-visible spectroscopy (ckx41) at 540 nm. The haemolysis rate (%) was calculated as:

$$\text{haemolysis rate (\%)} = \frac{OD_x - OD_{PBS}}{OD_{H_2O} - OD_{PBS}} \times 100\%$$

where $OD_X$ denotes the absorbance of erythrocytes exposed to different groups of

sample mixture, negative control, and positive control, respectively.

**In vitro antibacterial activity**

Staphylococcus aureus, Escherichia coli was diluted with nutrient broth to a final concentration of $1\times10^6$ CFU/ml for antibacterial investigations. The electrode materials were cut into 1×1 cm size, soaked in alcohol for 30 min, then UV for 30 min and placed in glass tubes containing 2 mL $1\times10^6$ CFU bacterial solution, and incubated in a carbon dioxide bacterial incubator at 37 ℃ for 24 h. After 24 h, the bacterial solution was removed, diluted 10-fold, and then tested for the OD at 600 nm for each group of bacterial solution. The remaining bacterial solution was diluted $5\times10^3$ times, and 300 μL of bacterial solution from each group was applied on sterile agar petri dishes and incubated at 37 ℃ for 24 h. The bacterial inhibitory activity was detected. The bacterial survival rates of S. aureus were calculated by using the following formula:

$$\text{bacteria survival (\%)} = \frac{C}{C_0} \times 100\%$$

where $C_0$ is the concentration of the bacteria before treatment and C is the remaining concentration of the bacteria after the treatment.

**In vivo wound healing test**

All animal experiments were approved by the Institutional Animal Care and Use Committee (No. 109037) and conducted according to the guidelines of the Animal Protection Act of the Board of Agriculture. 8-week-old Kunming male mice (45 ± 5 g) were purchased from Liaoning Changsheng Biological Co. and were randomly divided into different groups with n = 4 mice per group. The mice were anaesthetized intraperitoneal with 1.25% tribromoethanol (Aphrodite) at a dose of 0.2 ml/10 g. After fixation of the mice, the backs of the mice were shaved and sterilized with alcohol. A biopsy perforator was used to create a full circular wound (8 mm in diameter) in the dorsal region of the mice, and the mice were housed in separate cages after the operation. The control group was covered with gauze only. The experimental group was treated with electrical stimulation, the electrode material was covered on the wound surface and closely fit with the traumatized skin, connected to the power supply, and electrical stimulation of 1 V was applied for 30 min, and the time of modelling was set to be day

0 (untreated), then the mice in both groups were given the corresponding treatments every day. Treatments were given every other day after the mice had scabbed over their wounds. Wound closure was monitored at different time intervals by taking photographs on days 0, 3, 6, 9, 12, and 15, and the mice were monitored daily for weight changes. Wound healing was quantified by calculating the wound area using Image J software and was expressed as follows:

$$\text{Wound/contraction area}(\%) = \frac{M_t}{M_0} \times 100\%$$

where $M_0$ and $M_t$ are the wound area at day 0 and the remaining wound area at different time intervals (days 3, 6, 9, 12 and 15), respectively.

**Immunohistochemically analysis**

All mice were executed after 15 days of treatment, and the traumatized tissue, including about 5 mm of normal skin near the wound, was exfoliated, fixed in 10% formalin buffer for 24 hours at room temperature, paraffin-embedded, and prepared for tissue sectioning. Vertical sections were cut along the center of the wound and stained with H&E and Masson stains, respectively. Representative sections from the treatment and control groups were imaged using an inverted microscope and analyzed using Image J. The slices were then stained with H&E and Masson stains, respectively.

**Evaluation criteria**

At the cellular level: Compared to the control group, the ES group should demonstrate a ≥30% increase in scratch healing rate within 24 hours and a ≥20% increase in proliferation rate.

Animal Level: On Day 7 of treatment, the residual wound area in the ES group should be significantly lower than that in the control group (target <40%).

**Part 1: Characterization and analysis of materials**

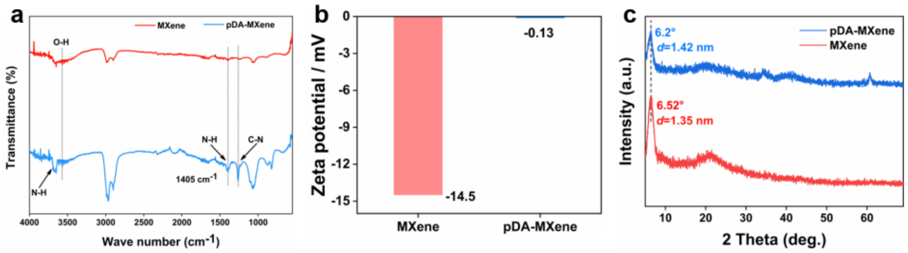


**Figure S1.** (a) FT-IR, (b) Zeta potential and (c) XRD profile of MXene and pDA-MXene.

Note: The supplementary FT-IR spectra show that compared to pure MXene, pDA-MXene exhibits a new absorption peak at 1405 $cm^{-1}$, which is attributed to the N-H in-plane bending vibration of the indole ring in pDA, and a C-N stretching vibration peak near 1280 $cm^{-1}$. After DA modification, the Zeta potential of MXene (-14.5 mV) changes to -0.13 mV, indicating a reduction in electronegativity, which is due to the introduction of positively charged groups such as amines in pDA. After modification with DA, the d-spacing between MXene nanosheets increased from 1.35 nm to 1.42 nm, indicating that the adhesion on DA surface effectively prevented severe surface-to-surface overlapping stacking of MXene nanosheets.

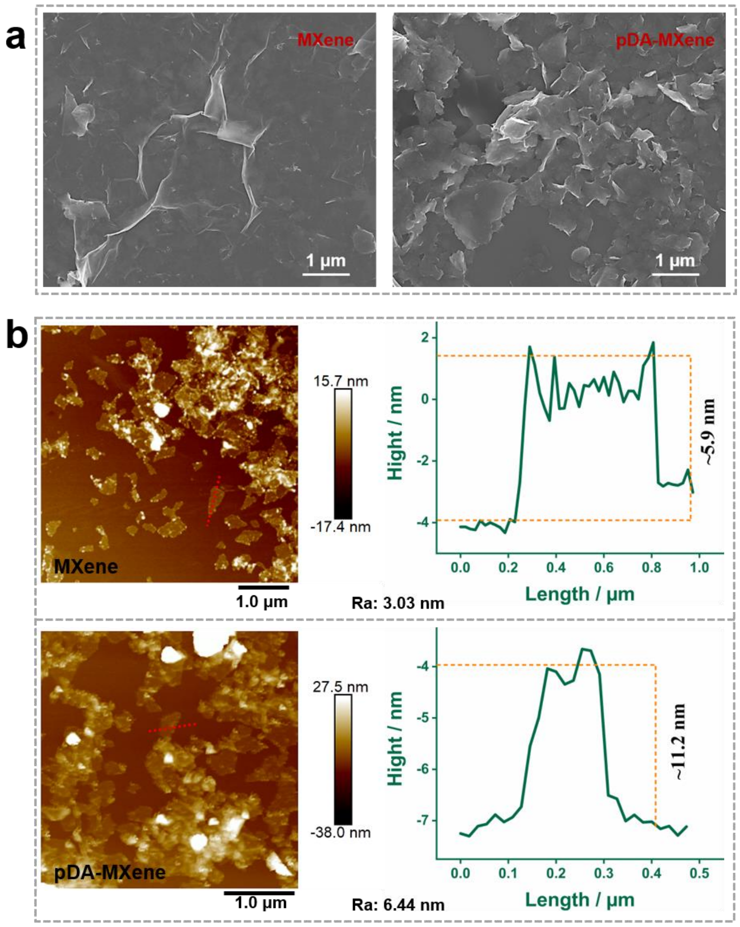


**Figure S2.** (a) SEM image of MXene and pDA-MXene, (b) AFM images and the corresponding height profiles suggest the surface roughness of MXene and pDA-MXene.

Note: The morphology of materials was characterized by SEM and AFM. The results showed that pure MXene exhibited a typical 2D nanosheet structure with clear edges and smooth surface (roughness: 3.03 nm). After DA modification, the surface roughness of pDA-MXene significantly increased to 6.44 nm, with uniformly distributed granular or fuzzy coating observed, and the edges tended to become blurred. Intercalation between nanosheets often became more compact due to the adhesive effect of pDA, or continuous coating layers were formed, and the apparent thickness of the nanosheets also generally increased (MXene: ~5.9 nm; pDA-MXene: ~11.2 nm). This morphological change is direct evidence of the successful polymerization and coating of dopamine on MXene surface.

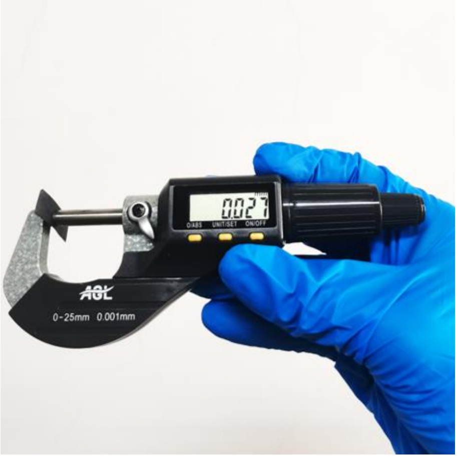


**Figure S3.** Thickness measurement of PMAB.

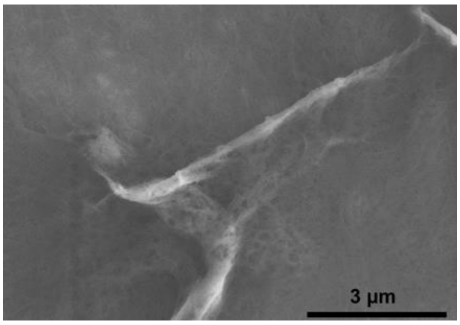


**Figure S4.** SEM image of BC network.

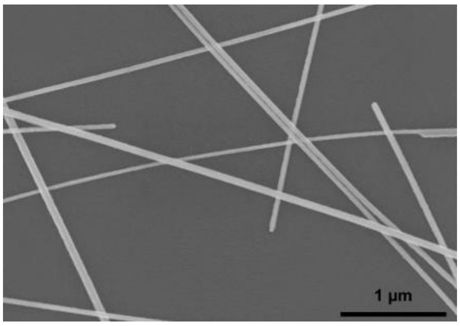


**Figure S5.** SEM image of Ag NWs.

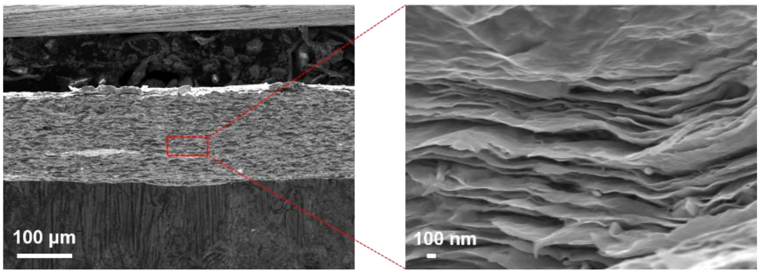


**Figure S6.** Cross-section SEM image of MXene nanosheets.

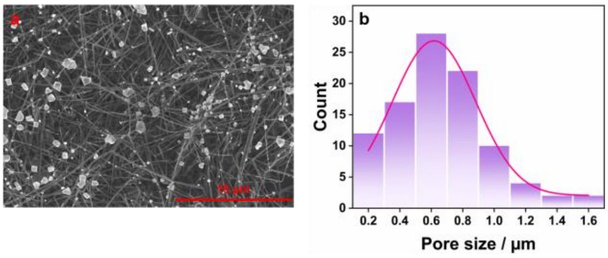


**Figure S7.** SEM image (a) and pore size distribution (b) of PMAB.

Note: Using ImageJ software and porosity formula (1), the porosity was determined to be approximately 31.7%. However, fibroblasts typically exceed 30 μm in length [1]. Given the current pore dimensions of PMAB, there are insufficiently large channels for cellular passage.

$$Porosity\ (\%) = \frac{Pore\ area}{Total\ area} * 100\% \qquad (1)$$

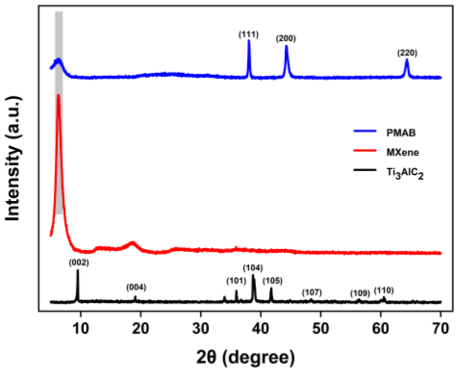


**Figure S8.** XRD pattern of MAX phase, MXene film, PMAB film, respectively.

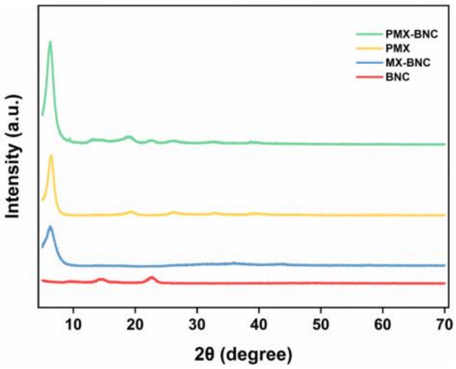


**Figure S9.** XRD pattern of MAX phase, MXene film, PMAB film, respectively.

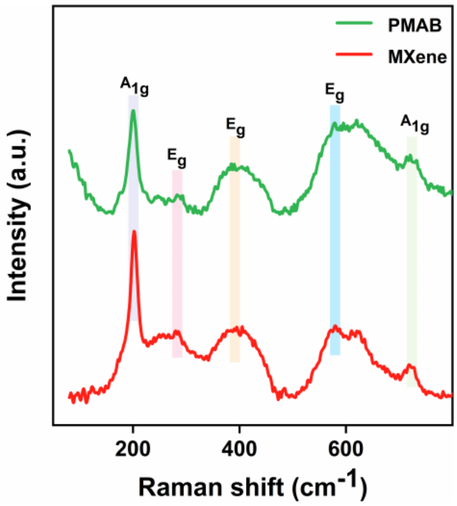


**Figure S10.** XRD patterns of BC (red line), MX-BC (blue line), PMX (yellow line) and PMX-BC (green line).

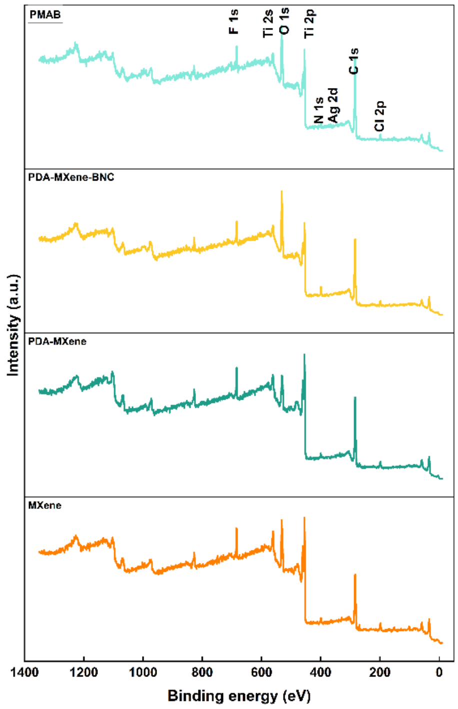


**Figure S11.** XPS survey of MXene, pDA-MXene, pDA-MXene-BC and PMAB.

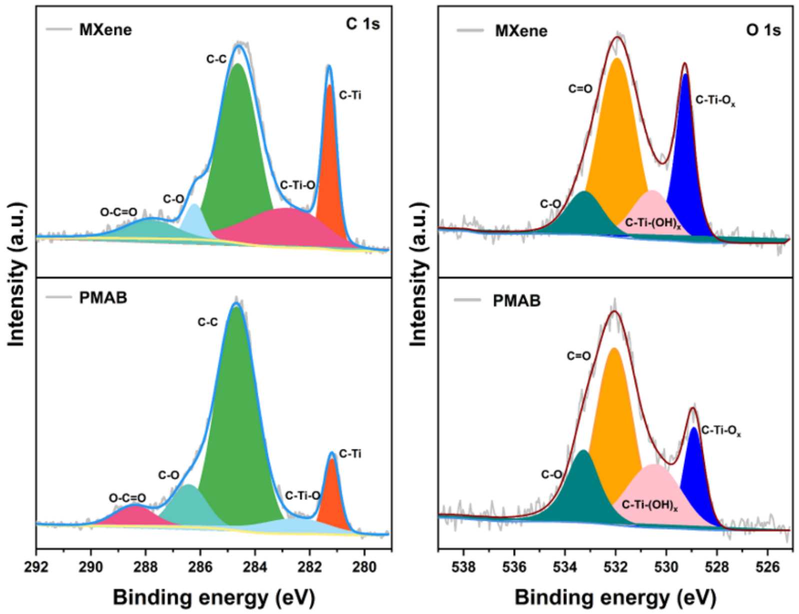


**Figure S12.** XPS spectra of C 1s and O 1s of PMAB and MXene.

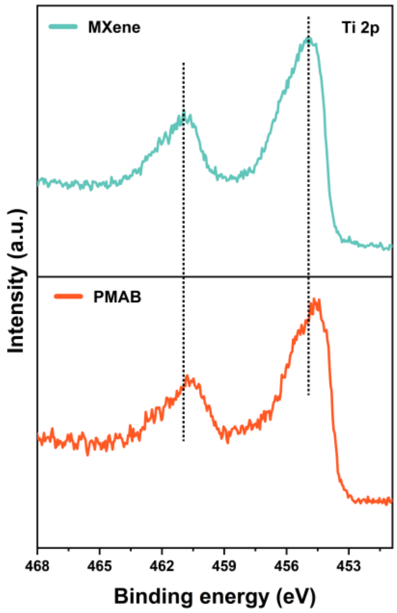


**Figure S13.** Comparison of high-resolution XPS spectra of MXene (green line) and PMAB (orange line) of Ti 2p.

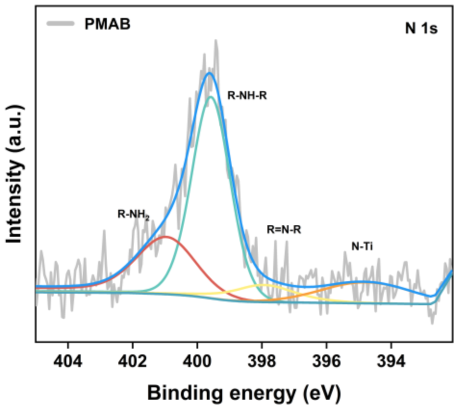


**Figure S14.** High-resolution N 1s XPS spectra of PMAB.

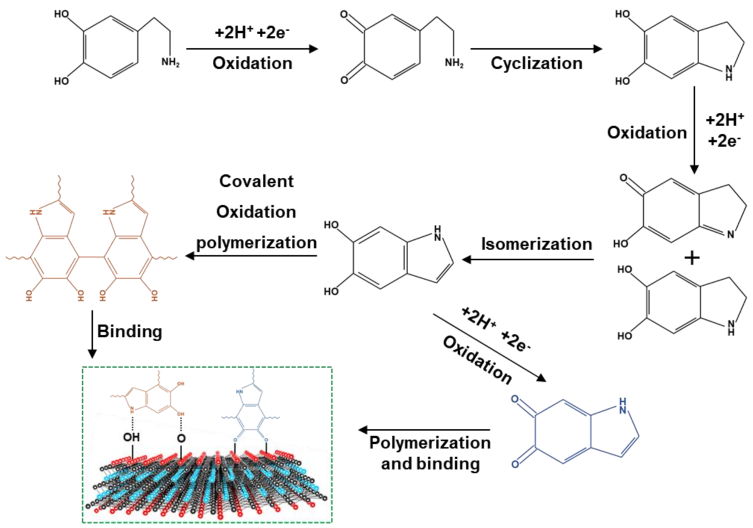


**Figure S15.** Oxidative polymerization of dopamine monomers and the binding stoichiometry of pDA to MXene.

**Part 2: Electrochemical perfoemance of the PMAB-based mocro-supercaoacitor**

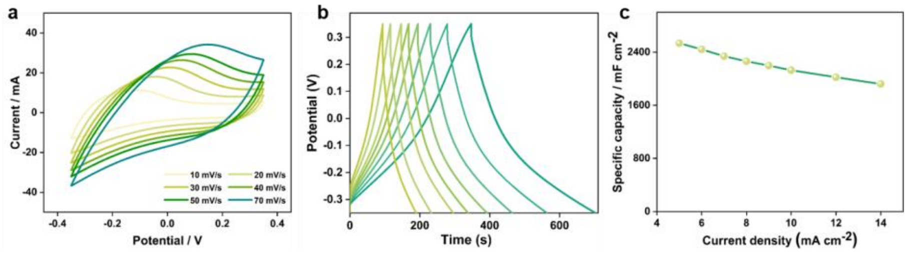


**Figure S16.** CV curves (a) and GCD profiles (b) of PMAB-based MSCs. (c) Specific capacitances at different current densities of PMAB electrode.

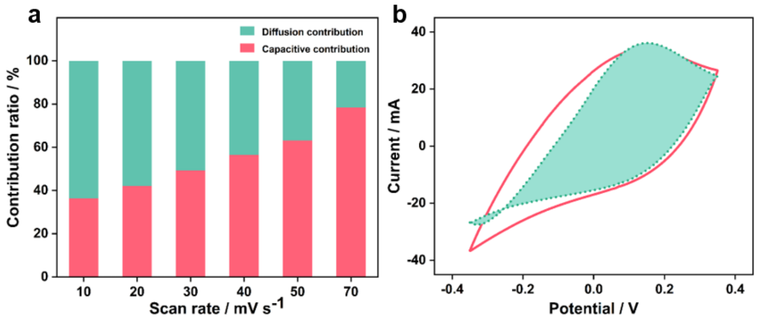


**Figure S17.** (a) Capacitance contributions under different scan rates. (b) Capacitive-contribution ratio of PMAB electrode at 10 mV $s^{-1}$.

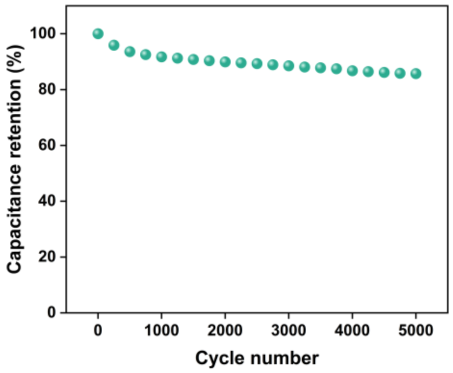


**Figure S18.** Cycle stability of PMAB electrode after 5000 cycles.

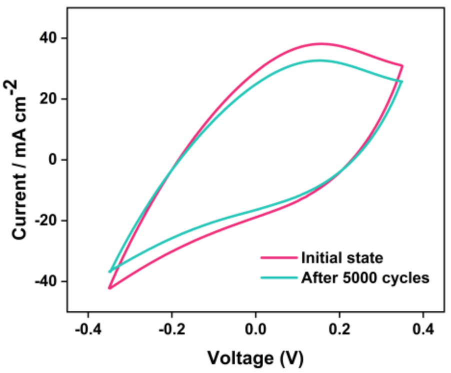


**Figure S19.** Comparison of CV curves of PMAB electrode after 5000 cycles.

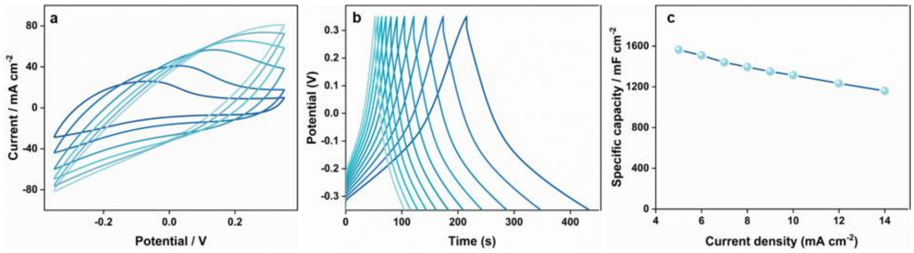


**Figure S20.** Electrochemical behavior of pure MXene electrodes. (a) CV curves at various scan rate. (b) GCD curves and (c) specific capacitance at different current density.

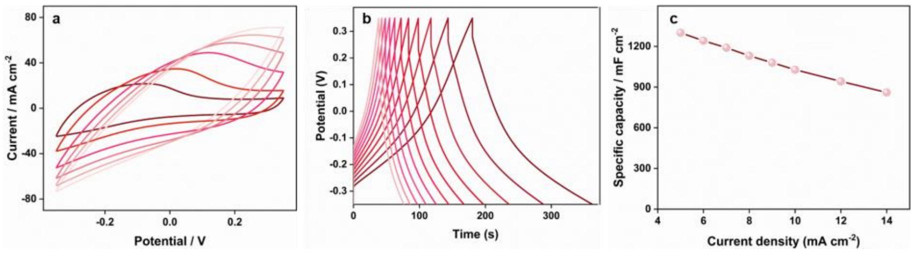


**Figure S21.** Electrochemical behavior of MXene-BC electrodes. (a) CV curves at various scan rate. (b) GCD curves and (c) specific capacitance at different current density.

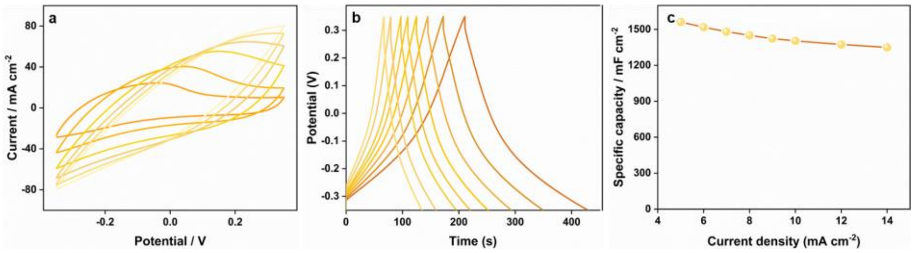


**Figure S22.** Electrochemical behavior of pDA-MXene electrodes. (a) CV curves at various scan rate. (b) GCD curves and (c) specific capacitance at different current density.

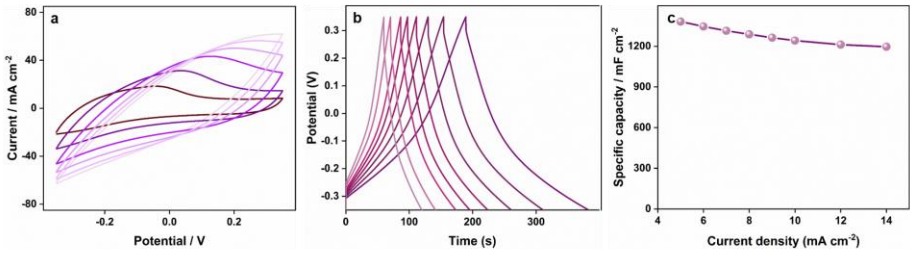


**Figure S23.** Electrochemical behavior of pDA-MXene-BC electrodes. (a) CV curves at various scan rate. (b) GCD curves and (c) specific capacitance at different current density.

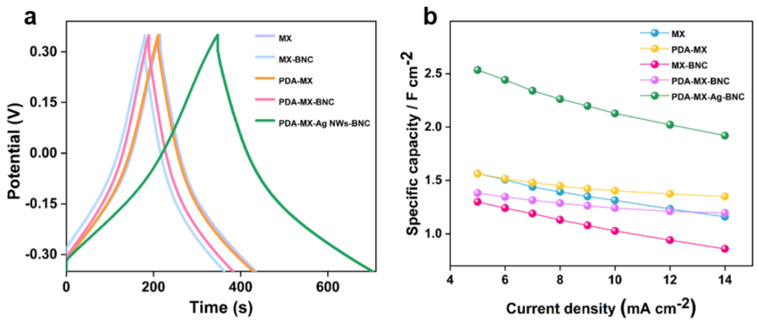


**Figure S24.** (a) GCD profiles of MXene, MXene-BC, pDA-MXene, pDA-MXene-BC and PMAB electrode at 5 mA $cm^{-2}$. (b) Specific capacitances at different current densities of MXene, MXene-BC, pDA-MXene, pDA-MXene-BC and PMAB electrode.

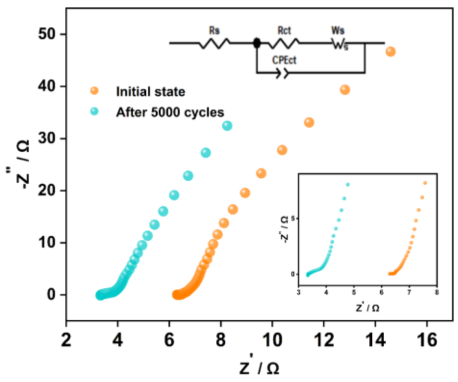


**Figure S25.** Nyquist plots of PMAB electrode after 5000 cycles.

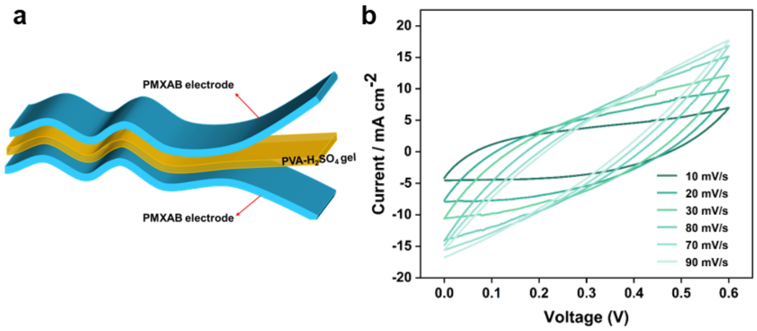


**Figure S26.** (a) Schematic diagram of assembled PMAB-based all-solid-state SCCs. (b) CV curves of PMAB-based all-solid-state SCCs with different scan rate.

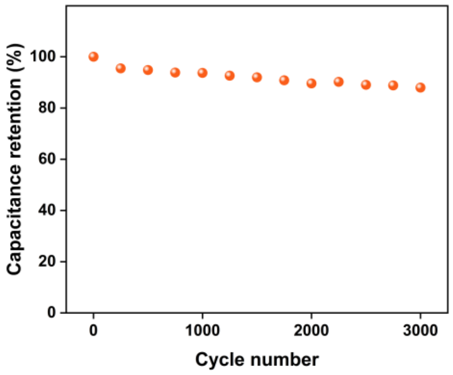


**Figure S27.** Cycle stability of PMAB-based SCCs after 3000 cycles.

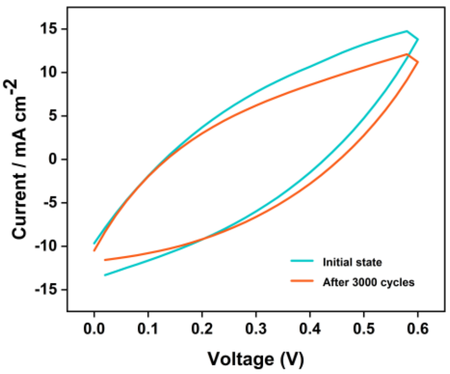


**Figure S28.** Comparison of CV curves of PMAB-based SCCs after 3000 cycles.

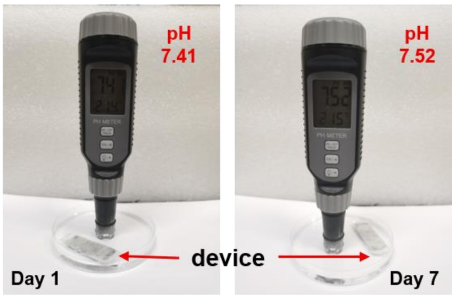


**Figure S29.** pH measurements of assembled supercapacitors over different days in pH 7.4 PBS.

Note: The encapsulated devices were immersed in PBS at 37 ℃ for 7 days, followed by detection of pH value. The results showed no significant change in pH demonstrating effectiveness of the encapsulation.

**Part 3: Cell biocompatibility and antibacterial testing**

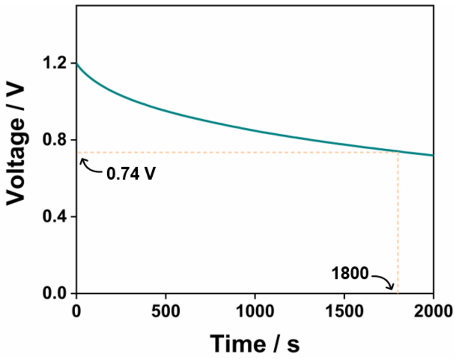


**Figure S30.** Self-discharge curve of PMAB-based SCCs.

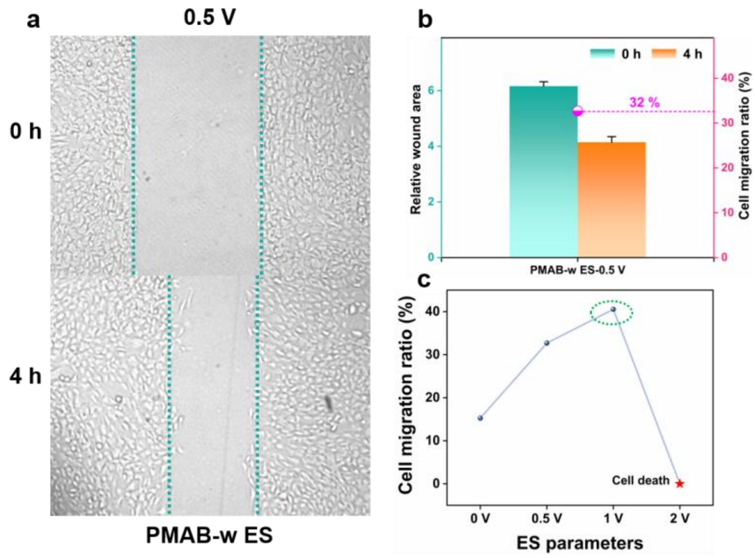


**Figure S31.** (a) Cell migration of NIH 3T3 cellular of PMAB-w/ ES-0.5 V groups at 0 and 4 h. (b) Corresponding cell migration ratio at 0.5 V. (c) Relationship between ES dose and corresponding cell migration rate.

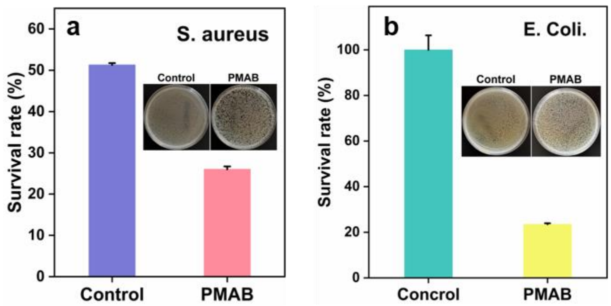


**Figure S32.** Bacterial survival ratios of (a) *S. aureus* and (b) *E. coli* for the control group. (Inset of digital photos of bacteria (*E. coli* and *S. aureus*) clones on culture plates after contacting with PMAB).

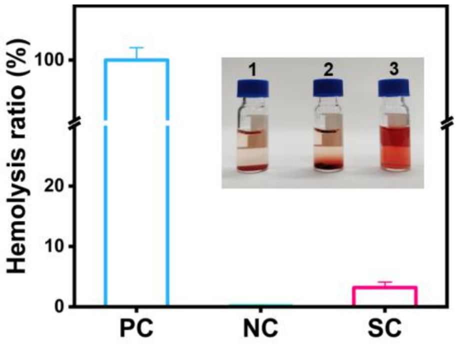


**Figure S33.** Hemolysis test for the PMAB-based electrode. 1#: RBCs+PBS (negative control group, NC); 2#: RBCs+PBS+3×3 mm sample (sample group, SC); 3#: RBCs+$H_2O$ (positive control group, PC).

**Part 4: Wearable PMAB-based epidermal pressure sensors performance**

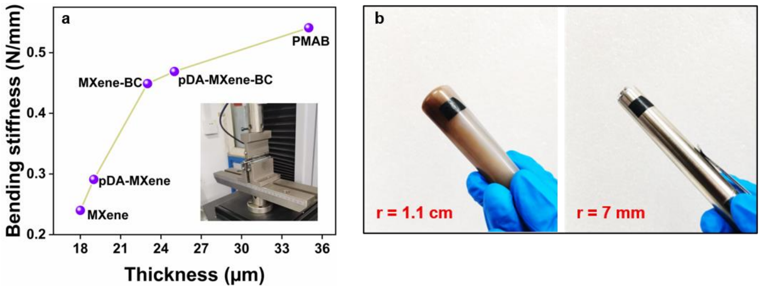


**Figure S34.** (a) Relationship between bending stiffness and thickness. (b) Minimum bending radius test.

Note: The relationship between thickness and bending stiffness for five materials (MXene, pDA-MXene, MXene-BC, pDA-MXene-BC and PMAB) was investigated by bending tests. As shown in Figure 1, materials thickness increased with the degree of modification enhanced. A three-point bending test measured the flexural stiffness of a 35μm-thick PMAB film at approximately 0.541 N/mm (Figure S33a).

Its minimum bending radius reached ~7 mm without cracking or conductive layer peeling (Figure S33b). When applied to cylindrical surface simulating a finger or wrist joint, the material achieved wrinkle-free conformal contact. We consider this a critical factor for maintaining a stable, low-impedance skin-electrode interface.

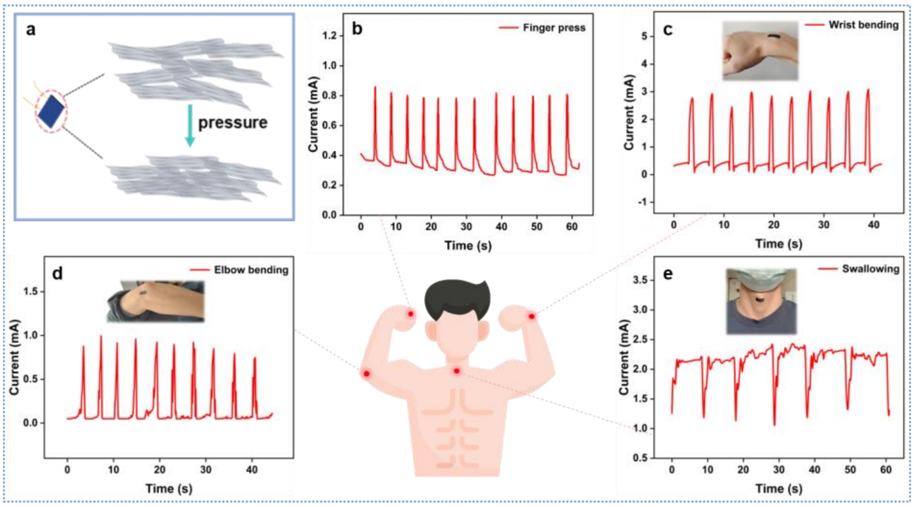


**Figure S35.** Evaluation of epidermal sensing performance of PMAB-based pressure sensors. (a) Sensing mechanism illustration of PMAB-based epidermal pressure sensors. (b) Response to consecutive press and release of a finger. Response to wrist bending (c), elbow bending (d), swallowing (e).

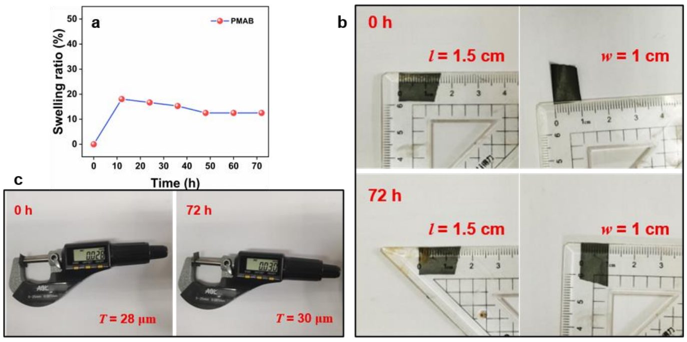


**Figure S36.** Water absorption rate (a), area swelling rate (b) and thickness swelling rate (c) over time of PMAB in 0.1 M pH 7.4 PBS.

Note: To evaluate the stability of PMAB dressings in body fluid environments, we systematically measured their water absorption rate, area and thickness swelling rate. Thoroughly dried samples (1.5×1 cm) were immersed in 37℃ PBS until swelling equilibrium was reached. Changes in mass, area, and thickness before and after swelling were measured and calculated separately. The specific formula is:

$$Water\ absorption\ rate = \frac{m_n - m_0}{m_0} \times\ 100\%$$

$m_n$ is final weight, $m_0$ is initial weight;

$$Area\ Swelling\ Rate = \frac{(l_s \times w_s) - (l_0 \times w_0)}{l_0 \times w_0} \times\ 100\%$$

$l_s$ and $w_s$ is final sizes, $l_0$ and $w_0$ is initial sizes;

$$Thickness\ Swelling\ Rate = \frac{T_n - T_0}{T_0} \times\ 100\%$$

$T_n$ is final weight, $T_0$ is initial weight.

After 72 hours of immersion, PMAB exhibited swelling rate of approximately 13% while maintaining its structural integrity. This demonstrates that PMAB network possesses excellent hydrophilicity (Figure S35a). However, PMAB retained its original area and structural integrity, demonstrating PMAB network's exceptional mechanical strength and stability (Figure S35b). Finally, thickness expansion rate of PMAB is approximately 7% (Figure S35c).

These results indicate that crosslinked interpenetrating network structure formed by BC fiber network and MXene layers undergoes strong in-plane constraints (e.g., hydrogen bonding interactions) in X-Y plane, limiting its planar expansion. Conversely, in the thickness direction (Z-axis), network can expand relatively freely to accommodate more water molecules, resulting in more pronounced thickness swelling.

Reference:

[1] Z. Qin, R.M. Balimunkwe, T. Quan, *British Journal of Dermatology*, **177** (5), 1337-1348 (2017).
